# Application of discrete choice experiments to estimate value of life: a national study protocol in Iran

**DOI:** 10.1186/s12962-021-00259-7

**Published:** 2021-01-30

**Authors:** Negar Mirzaee, Amirhossein Takian, Farshad Farzadfar, Rajabali Daroudi, Ali Kazemi Karyani, Ali Akbari Sari

**Affiliations:** 1 Department of Health Management and Economics, School of Public Health, Tehran University of Medical Sciences (TUMS), Tehran, Iran; 2 Department of Global Health and Public Policy, School of Public Health, Tehran University of Medical Sciences (TUMS), Tehran, Iran; 3 Health Equity Research Center (HERC), Tehran University of Medical Sciences (TUMS), Tehran, Iran; 4 Non–communicable Diseases Research Center, Endocrinology and Metabolism Research Institute, Tehran University of Medical Sciences (TUMS), Tehran, Iran; 5 Department of Public Health, School of Health, Kermanshah University of Medical Sciences, Kermanshah, Iran

## Abstract

**Background:**

Global concerns regarding the significant burden of non-communicable diseases and injuries (NCDIs) exist from both public health and economic perspectives. Our research focuses on the reduction of fatal risks due to NCDIs and the citizens’ preferences about health programs and intervention to reduce premature death due to NCDIs. Governments and health authorities need reliable evidence and information to prioritize the interests of their citizens. One crucial piece of evidence to justify the resources spent on NCDIs is the value derived from the interventions on prevention and NCDIs control. This concept is usually called “Value of Statistical Life” (VSL), meaning the monetary value that individuals place on changes in the risk levels of life- threatening events. To the best of our knowledge, for the first time, our study will estimate the statistical value of life for selected interventions for the prevention and control of NCDIs at both national and sub-national levels in the context of Iran. This paper reports the development of a national protocol through Discrete Choice Experiments (DCEs) method.

**Methods and designs:**

Our study comprises several stages: (a) a literature review to identify the attributes and levels of the prevention programs and Willingness to Pay (WTP) for reducing the NCDI’s fatal risks; (b) experimental design to assessing, prioritizing, and finalizing the identified attributes and levels; (c) instrumental design to conduct face-to-face structured survey interviews of 3180 respondents aged 18–69 across the entire country; (d) statistical analysis to estimate the results through the Mixed Multinomial logit (MMNL) model.

**Discussion:**

We anticipate that our findings will help build a stronger empirical basis for monetizing the value of small changes in selected fatality risks. It paves the way for other national or vast VSL estimates for NCDIs, as well as other major causes of morbidity and mortality in the context of Iran, and perhaps other low and middle-income countries (LMICs).

## Background

Mortality and prolonged disability related to NCDs have considerable economic impact on households and industries, through the application of health services and losses in income, productivity and capital formation [1, 2]. Along with other 193 member states, Iran is committed to reach the United Nations’ sustainable development goals (SDGs). SDG 3.4 aims to reduce 30% premature death associated with non- communicable diseases (NCDs) globally, i.e. in Iran [3]. Understanding the appropriate amount of resources to health and safety programs allocated by government requires an understanding of the value that individuals place on changes in risk levels for life threats and health problems.

Therefore, over the past three decades, many governments and affiliated agencies have been adopting the Value of Statistical Life (VSL) approaches to value risk regulations [4, 5]. Despite many VSL studies in high-income countries (HICs), evidence from low and middle-income countries (LMICs), such as Iran is scarce. The VSL estimation is affected by the employed methods, risk reduction levels, risk reduction preferences, the demographic and economic characteristics of the study population. Some studies indicated that using a single VSL is inappropriate for all policy benefits of and all deaths Therefore, direct VSL estimation is essential for a country of concern. Since there is a growing concern about the significant burden of non-communicable diseases and injuries (NCDIs) from both public health and economic perspectives. Our research focuses on fatal risk reduction due to NCDIs, which is associated to 81% of premature death in Iran [6]. Therefore, understanding VSL is vital to determine the value of public policies designated to protect humans’ life, promote health, and avoid preventable death. VSL is the marginal rate of substitution between income and mortality risk. This concept indicates how much individuals are willing to pay (WTP) to reduce the risk of death, which was presented in one study as willingness to swap (WTS_μr_) for a micro-risk reduction in the chance of death (or other types of risks to life and health) [7].

To achieve VSL in the LMICs, there are three practice lines (effective activities): Scaling, meta-analysis and direct estimation [8–10]. The scaling approach is based on the estimated value of VSL in high-income countries (HICs), which is estimated by calibrations based on the income differences [11]. Since in this approach, the VSL is determined only by the level of per capita income and the income elasticity in countries is not constant, adopting another approach is inevitable [12]. The VSL meta-analysis approach in the HICs is used to predict the VSL estimates in the LMICs by considering the differences in risk, income, level of human capital, and demographic characteristics [13]. As most studies have been performed on the HICs, this approach might be unreliable due to the inconsistencies among specific factors in the LMICs and some assumptions made in the original studies of HICs [8].

Two methods can be used to estimate the direct VSL [1]: human capital (HC) and WTP. The HC approach places monetary weights on healthy time using market wage rates and the value of the program is assessed in terms of the present value of future income [14–16].

The WTP approach is based on publics’ preferences as a measurement of increasing in human well-being due to risk reduction of mortality. The WTP approach therefore is often called the preference-based approach categorized in two types [15–18]. The first type is revealed preference (RP) or observed preference, which is measure for understanding the value that individuals place on goods and services in a real market [14–19]. The RP approach is used to determine the monetary value in two ways: (a) the use of hedonic methods (including hedonic prices, hedonic wages) and shadow prices, (b) purchasing behavior (Averting Cost or Self-protection) [20–22].

**Fig. 1:**
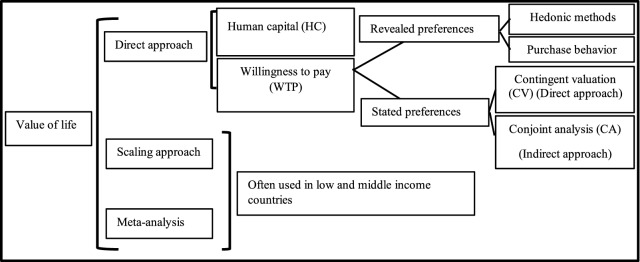
The categories of VSL estimation methods

The second WTP approach is stated preference (SP), which uses survey methods to present respondents with hypothetical scenarios about the program or problem under evaluation [15]. Two methods can be used to elicit the stated preferences, including: contingent valuation (CV) or direct approach and conjoint analysis (CA) or indirect approach [14]. In the CV method, individuals are asked to directly express their maximum willingness to pay for a product or service contingent in the market. The CA approach is a general name to show individuals are willing to trade between the characteristics of interventions, to estimate the relative importance of different attributes, to estimate whether an attribute is important, and to predict the demand for a given good or service with given attributes [14]. Discrete choice experiment (DCE) is a type of CA that presents two (or more) scenarios to the respondents and asks them to choose the one that they prefer [23, 24]. The summary of VSL estimation methods is illustrated in Fig. 1.

In this study, we will perform the first VSL estimation in Iran using the DCE method. Health system planning has been conventionally skewed towards the supply side [25]. This study will be an innovative attempt to also take the demand side into considerations while allocating required resources to tackle NCDIs, which are supposedly the main cause of premature death in the country [26]. Since few VSL studies have been conducted in the context of the LMICs, our findings will pave the way, we envisage, better planning to reach SDG 3.4 in similar settings.

We report the methodological steps that we are going to take to estimate the VSL for the selected NCDIs in the Iranian context. Among the major NCDs, cardiovascular diseases and cancer are the top two causes of death in Iran. Moreover, road traffic injuries, as one of the main targets of SDG 3, are associated with 60% of all road traffic injuries death in Iran. Therefore, we included them in our study [27].

## Objectives of study

This paper has three purposes: to introduce the various steps we have taken to design an appropriate methodology for estimating VSL in Iran; to describe each methodological step in detail; and, to highlight the limitations and strengths of this methodological approach for future study considerations.

## Study design/methods

### Overview of the approach and method

This will be a Discrete Choice Experiment study. This approach has been employed in several disciplines and is increasingly used in healthcare. Initially, preparing a comprehensive literature review, we created a checklist to prioritize the identified features and levels to create selected collections in the next step. Second, we will form a specialized team consisting of relevant physicians, senior insurance and health system officials, as well as selected service providers to complete the study check list. Third, to determine VSL, we will conduct a stated preference survey among 3180 people. Finally, we will outline evidence-based policy recommendations to improve the resource allocation to combat NCDIs and its related risk factors in Iran The DCEs method is a quantitative approach to assessing medical intervention preferences, products, and policies. Qualitative study is also an integrated part of the DCEs development in order to identify relevant attributes and levels for the choice design. This method is based on Lancaster’s and Random utility theory [28, 29] and asks individuals to make decisions in a hypothetical situation. The method estimates the weights that respondents’ place on each of the characteristics, in a way to provide them with utmost satisfaction [30, 31]. The DCEs method is worth using when the intention is to establish the tradeoffs that people are willing to make for different attributes of the existing interventions [14, 32]. while several methods exists to do this [33, 34], we identified DCEs as the most appropriate method and used the International Society for Pharmacoeconomics and Outcomes Research (ISPOR) Guidelines for Good Research for conjoint analysis in this study [35, 36]. Below, we explain the four stages of the development process of DCEs in detail and summarize them in Table 1.

**Table 1:** Development of discrete choice experiments

Attributes and levels Identifications (potential attributes)
Literature Review: To investigate the attributes that are likely to be important to users
Selecting the essential attributes (removing the inappropriate attributes): expert interviews (experts panel)
Experimental design
Create proper tasks (choice sets): Orthogonal Fractional Factorial/ Blocking/ Imposed restrictions
Instrument design/data collection
To estimate WTP and value the risk reduction, a larger survey instruments will be needed: face to face structured administrated survey
Statistical analysis
Statistical analysis of data with logit model

### Step 1: identifying attributes and levels

To fulfill our research objectives, the most essential step is to identifying some of the characteristics that provide a practical description to evaluate and prevent the risk of premature death through selected NCDIs programs. We searched various databases including MEDLINE, PubMed, Web of Science, Scopus, Science Direct and Google Scholar engine for English studies since 1990. We also used Medical Subject Headings (MeSH) and keywords including discrete choice experiment (s), discrete choice model, conjoint analysis, conjoint measurement, conjoint studies, stated preferences, preferences elicitation, non-communicable disease (NCDs), prevention of cardiovascular diseases, road injuries,cancer, Life Valuation, Economic Life valuation(s), Economic Value of Life, Life Economic Value(s), Value of statistical life, Value of statistical life year(s), value of risk reductions. This process entails scoping literature review, which will provide us with a provisional list of some attributes. The findings will be used in later steps to finalize our DCE attributes and levels, which comprises the raw data collection [37]. A list of nine features was presented in this review, which was then reduced to a limited number of attributes during the next stage. An expert panel comprising of nine relevant key informants including clinicians and selected senior officials in the insurance and health system was convened to approve, select and reduce the final list of attributes, as well as to ensure that no potentially important attributes were overlooked in the final list. The session was audio-recorded and the investigator made observational notes during the meeting. As attributes should comprise all the elements relevant to an individual’s decision, the former key informants were approached in the process of preparing the list of these attributes through a free-of-charge trial of the Mentimeter Pro software and asked to finalize the list as well as weigh and rank the attributes and levels using a tailored Likert-scale questionnaire. The literature on what constitutes a controllable number is unclear, but most studies preferred a maximum of six or seven attributes to minimize the burden on respondents [38]. Ultimately, five attributes with a maximum of four levels were derived in the VSL context: (1) the cause of death; (2) the risk reduction plan or program; (3) effect starts will express as the effects of the programs which are commencing X years from the implementation date and should be continuing for 10 years; (4) the risk reduction itself; and (5) cost. Attributes and attribute levels are summarized in Table 2.

**Table 2:** Summary of attributes and attribute levels in the discrete choice experiments

Attributes	NO. levels	Levels
Cause of death	3	Cardiovascular diseases, Cancer, Road traffic accidents
Risk reduction plan	4	Reducing harmful use of alcohol and tobacco, Healthy diet, Physical activity, Effective road safety measures
Effect starts	4	Immediately, 2 years later, 5 years later, 10 years later
Risk reduction	4	3/10,000, 6/10,000, 9/10,000, 15/10,000
Cost	4	12, 24, 36, 48 million IRR per year (in Islamic Republic of Iran Rial, about 284, 569, 853,1137 US dollars)

### Step 2: experimental design

The alternatives in the DCEs are of the unlabeled type and have generic headings in a full fractional design with several combination of attribute levels. In our study, the experiment consisted of four attributes at four levels and one attribute with three levels requiring a profile of 4^4^ × 3^1^ or 768 (scenarios/runs) [39], which is equal to [(768*767)/2] or 294,528 choice sets. This usually leads to a large number of choice questions that are tedious for the respondents as well as expensive for investigators. Therefore, to observe practicalities, we used a fractional factorial design to construct the DCEs, which requires fewer runs compared with full factorial designs. To make our study design more practical and realistic, we also imposed restrictions on the attribute-level combinations (runs) [36, 39]. For example, the combination of cancer level as the cause of death attributes, and road safety measures level as the risk reduction plan attribute would be unrealistic. In this way, we managed therefore to avoid the implausible combinations.

**Fig. 2:**
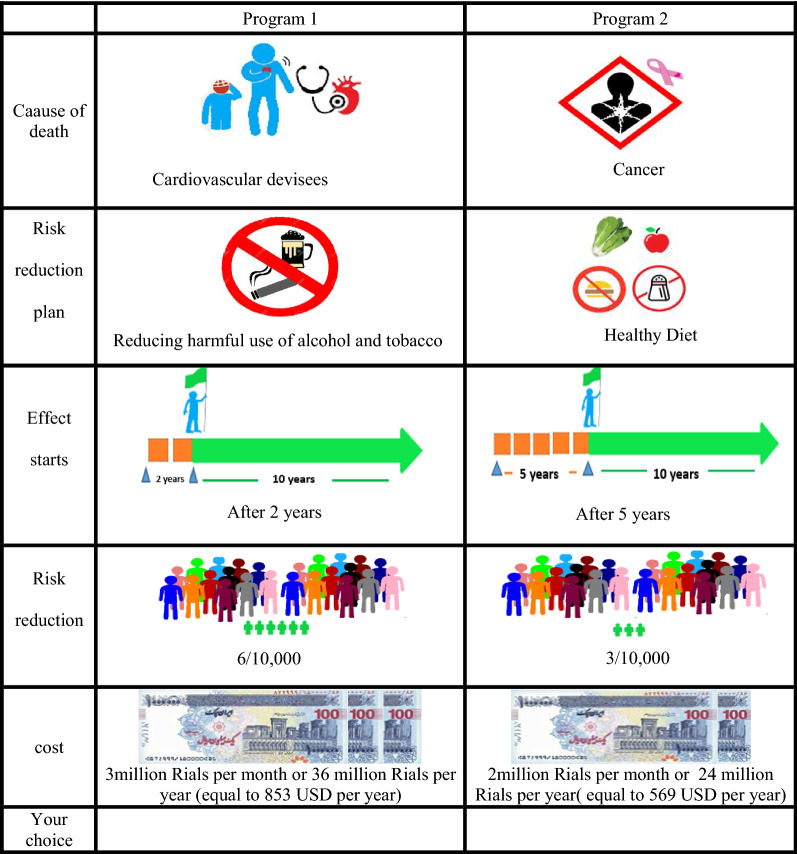
An example of a DCE choice set

We used SAS V.9.4 to provide a D-efficient design and reduce the number of runs [39], which reduced our choice sets to 40. To minimize the effects of low-response rate and the cognitive effects for each respondent to the survey, we categorized the choice sets into five blocks with eight choices per block [29, 36]. Each block will be randomly assigned to each respondent by the software. The choice sets will be explained as textual and visual scenarios. We will use visual elements to reduce the likelihood of possible dropping due to choice set complexity or illiteracy that will help participants to quickly identify the levels [23, 40, 41]. An example of a DCE choice task is shown in Fig. 2.

### Step 3: instrumental design

In this phase, we will use a face-to-face structured survey administered to general-population samples of 3180 individuals who will be registered in the STEPs (Surveillance of Risk Factors of Non–Communicable Diseases) 2020 study across Iran [33]. The eligible participants will be all households over 18 years old. Because we have designed this study to be conducted as a prospective research, we have reached the Ministry of Health & Medical Education (MoHME) of Iran and the Research center for NCDs affiliated with Tehran University of Medical Sciences (TUMS) in order to conduct our survey along with the national survey of NCDs and their related risk factors in Iran (STEPs). Unfortunately, due to COVID-19 pandemic, STEPs survey has been put on hold. We would sincerely hope that we will be able to conduct this study through ideal face-to-face interview during STEPs data collection of households. We still observe some considerations for both questioners and individuals (respondents) in COVID-19 pandemic to mitigate the risk of data collection: if the questioners and individuals are experiencing symptoms consistent with COVID-19, including fever, chills, cough, shortness of breath, or sore throat in the last two days, the questioners should be changed and the individuals will be banned to attend. Therefore, the questioners should ensure that all individuals are being asked to disclose information about cold or flu symptoms. To protect the questioners and participants, we have developed a COVID-19 plan that you can be 1.5 m away from others wherever possible; set up interview sessions outdoor and in the open air if possible, open windows, or adjust air conditioning for more ventilation; and wear surgical mask and, if possible, a face shield. The questionnaire includes an introduction, some explanations about the importance of participants’ role, the consent form, questions about screening risk as well as respondents’ demographic and behavioral information, explanations of choice sets and finally 10 core choice sets. The questionnaire consists of eight different choice sets and one dominance choice set, which is a completely superior program and helps us examine the rationality of the participants' selection behavior [42]. The survey will begin with three screening risk questions related to the risk comprehension questions. Participants who will fail to respond two out of the three risk questions will be excluded from the survey, aiming to avoid the inclusion of distracted or unconsidered participants who would likely crop inconsistent data. Moreover, the questionnaire will include two intentionally repetitive choice sets to check the reliability of the eight main choice sets.

Considering the possibility of overestimation of VSL (risk of perception), we have embedded some score of risk perception variables. Literature shows that some factors influence a person’s WTP for improvements in saving lives, e.g. the perception of risk. In this regard, both a general understanding of risk and social values are effective for risk change [43, 44]. Economists and decision theorists have investigated the social value of health risk changes (e.g. the value of life) that should be employed in cost—benefit analysis of risk management options. Normative economic analyses have emphasized the importance of an individual’s wealth and the initial level of risk that influence WTP for reductions in risks [45]. Psychologists, on the other hand, have studied how people perceive and react to various dangers. Regarding this issue, three possibilities arise: (1) the concept of risk (respondents familiarity with the concept of risk and probability, etc.), to this; the research team provided the first three questions for people’s understanding; (2) the extent of knowledge about real risk of death due to NCDIs, that the researcher provided actual information about risk of death; (3) subjective perception of a specific risk, which we asked the respondents to rate their subjective perception of each risk by scoring on a Likert scale. For example, people with dread, fear, exposure or controllability, etc. might be willing to commit more resources to reduce risks, with which they are not familiar and/or they consider outside of their own control.

We reviewed the theoretical (content) validity of the questionnaire for all items or questions, except for the choice tasks, namely the Content Validity Index (CVI) and the Content Validity Ratio (CVR). The expert panel for finalizing the attributes had 13 experts. The Lawshe’s method suggests that items with a CVR of 0.54 or higher could be reflected evidence of good content validity [46–48]. If an item does not reach this threshold, it would generally be deleted from the final tool. The threshold of CVI is 0.7, while our CVI was 0.84, and we dropped only one item. Checking the validity of the content in the selected DCE collections means that the results are in line with prior expectations, which are assessed by examining the coefficient marks and their significance.

**Fig. 3:**
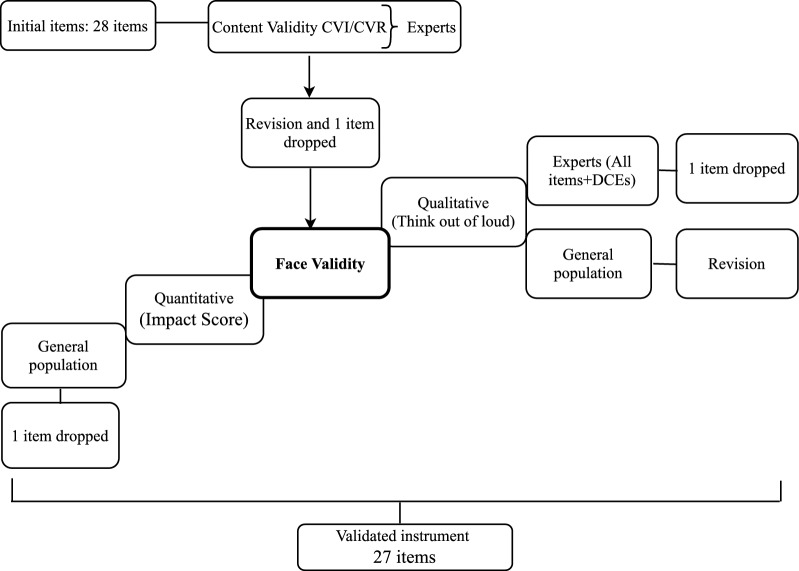
Stages taken for validating the questionnaire

Face validity was assessed through both quantitative and qualitative approaches. Qualitative face validity of all questions was assessed through interviews with 10 people from both target groups, i.e. the public and expert panels [46, 49]. To check qualitative face validity, we adopted ‘think out loud’ technique (interviewers will encourage participants to verbalize thoughts while answering the questions) [49], meaning that we asked 10 individuals from the target group to think out loudly when answering choice sets and all the questions [50]. This helped identifying respondents’ reactions, redundancies, irrelevant or unclear items, following which we then revised the questionnaire. The quantitative face validity was also checked for all questions except the choice tasks, using a five-point Likert scale and measure impact score. Figure 3 illustrates the validation phases of the final questionnaire.

To check reliability, the inter-rater reliability is investigated. Since we will conduct this study at both national and sub-national levels, we need to train many interviewers at different levels across the country. The inter-rater reliability was 0.7–1.0. The intra reliability is examined by including the same choice set twice in any given block. In addition, a pilot survey was conducted to review the complexity of the survey instrument and experimental tasks. Our comprehensive protocol developed based on Information Technology standards and guidelines to increase and improve the data registration and transmission accuracy. To control the quality and optimize the management of processes, we have developed an online Android-based tailored application, to be installed on tablets for data gathering, which will be also used for reporting and monitoring purposes. To increase the accuracy of data collection, we have created another application that reads tagged questionnaire and imports related data to database more quickly As data collection will be conducted within different provinces of Iran simultaneously, the project management team will monitor and evaluate all activities including data entry, data cleaning and training the interviewers in hierarchical steps at different levels. We have designed a comprehensive training package for interviewers, conducted several Training of Trainers (TOT) sessions [51], established a social media channel for direct and constant communication between the interviewers in all settings and the research management team as well as among interviewers to facilitate dialogue and optimize process management.

### Sample size

We will conduct our research at both national and regional levels in 31 provinces as representative samples of Iran. The appropriate sample size depends on the question format, the complexity of the choice tasks, the desired accuracy of the results, the degree of heterogeneity in the target population, the availability of respondents, and the need to conduct subgroup analysis [52]. There are certain ways to estimate the appropriate sample size for DCE studies [53]

(1) Johnson recommended this equation $\frac{\mathrm{nta}}{\mathrm{c}}\ge 500$ to estimate minimum sample sizes for the modeling of DCEs, where n is the number of respondents, t the number of tasks, a the number of alternatives per task, and c the largest number of levels for any one attribute (if we do not have interactions) [52]. It would be better that 1,000 representations should be in place rather than 500 per main-effect level [54, 55].

(2) The practical guidelines for the sample size recommend the appropriate size is 150–1200 [54]. As our aim was to compare the stratum, enough samples were needed to accommodate at least about 200 people in each group. To guarantee a robust quantitative research and a proper representation of the Iranian society, the sample size is 500 pre-strata or groups [54]. We have divided all 31 provinces in Iran into several strata stratified by the behavioral risk factors, employment and Wealth Index. This reached the total sample size to about 3000 [6 groups’ × 500] and 5% was added to estimate samples for control non-response error.

### Step 4: statistical analysis

The DCEs data, estimating VSL and modeling of preferences would require complex statistical analyses and methods. There are several objectives when analyzing DCEs data.

The first goal is to estimate VSL, the second is how it will differ in terms of features, and the third is to estimate preferences for the traits and levels in the survey. Another objective is estimating how preferences vary by individual respondent characteristics. And the last one is calculating the money equivalent, such as willingness to pay. Sensitivity analyses will be performed to determine the stability of the parameter estimates. When respondents face multiple choice sets, the MMNL model will be employed for the efficient design and suitable analysis model of the DCEs data [56, 57]. This model accounts for the correlation between unobserved utilities or random preference, as well as the panel effects and will be run by the Stata (v.14) statistical software. The marginal WTP for each attribute is obtained from the ratio of the estimated parameters of the attributes and the price, if the function of the attribute vector is linear. The results are reported as parameter estimates (β), or the 95% odds ratio and confidence intervals and their P values for the probability of selecting one option over another.

### Dissemination

As we have conducted the survey along with the National Survey of NCDs and their associated risk factors in Iran (STEPs), due to COVID-19 pandemic, STEPs survey has been put on hold. While the research team is committed to carrying out the project, we will conduct the research with the permission of the Ministry of Health & Medical Education (MoHME) of Iran and the Research center for NCDs affiliated with Tehran University of Medical Sciences (TUMS). We propose an eight-month-period for the rest of this project, including one month of preparations and logistics, four months for data collection and data monitoring, one month for econometrics analysis, one month for providing interpretation and policy analysis, publishing internal reports (holding various advocacy meetings, conferences, seminars, policy debates, and symposia for different target audience, i.e. policymakers, politicians, managers, etc.) and two months for submitting the results in scientific journals.

## Discussion

Iran is committed to achieve SDGs by 2030. SDG 3.4 pays special attention to the 30% reduction in premature death caused by NCDs, which by 2030 is associated with 81% of premature death in Iran [6]. Mortality and prolonged disability associated with NCDIs have considerable economic impact on households and industries, through the consumption of health services and losses in income, productivity and capital formation [1, 2]. Therefore, estimating the economic impact of NCDI is central to all settings, e.g. through VSL methods. Defined as the rate at which individuals are willing to tradeoff between income and risk [58, 59], understanding VSL is vital to determine the value of public policies designated to protect humans’ life, promote health, and avoid preventable death. To the best of our knowledge, this study is first of its kind to measure the value of life using the DCE method, specifically for the prevention and control of NCDIs in Iran, and most probably many LMICs, where the burden of NCDs is dramatically high [1, 5].. As the literature on public preferences for dealing with NCDI in the context of LMIC is scarce, we anticipate that our findings will help define evidence of informed political choices that align with public choices along the way to the SDG. 3.4. The ultimate impact of our research will be balancing the supply and demand sides of tailored healthcare interventions for prevention and control of NCDIs in Iran, and perhaps similar settings.

Our analysis will provide the estimation of the marginal effect (importance) of each attribute on the decision to select the prevention programs, for an instance, if a monetary attribute (cost/price) is untaken; the analysis will provide an estimate of relative importance of out of pocket cost on public’s decision for control and prevention programs. In addition to this analysis, the estimation of marginal rates of substitution between attributes, which be an indication of the extent to respondents, are prepared to trade-off one attribute for another, will be provided. For example, if the effect starts and the amount of risk reduction are offered as attributes, we will understand how the people are willing to accept a trade-off between latency of effect starts and more efficiency. As in our specific objectives, we will extract the general and public preferences of interventions to reduce the risk of mortality due to selected NCDIs, estimating the public’s willingness to pay for each feature to reduce the risk of death due to selected NCDIs; estimate the value of life in the Iranian context; and, provide evidence-based policy recommendations to include public preferences in defining the service packages for prevention and control of NCDIs in Iran. These allow policy makers to model the cost and better understanding of the trade-offs in doing so and ultimately deliver more effectively proven long-term strategies for the prevention.

Estimating mortality losses in the LMIC is important topic because many of these economies are in the epidemiological transition. Our study will provide extensive information on mortality valuation in developing countries and distinguish between HICs and LMICs, risk of death, age, education and other socioeconomic variables. Given the limited reliable information on health sector (increasing the level of exposure to risk factors for NCDs and the burden of NCDs) on LMICs, our future results will be a benchmark of VSL on LMICs settings.

The main results will contribute to: (a) understand the perceived cost of NCDs based on public preferences; (b) planning for the allocation of appropriate funds by Iran’s Ministry of Health and Medical Education, and Budget and Planning Organization for appropriate health interventions to reduce the burden of NCDs; and advocate the conduction of VSL studies by other governmental organizations and agencies, while taking various evidence-informed policies of concern.

## Abbreviations

CA: Conjoint analysis
CVI: Content validity index
CVR: Content validity ratio
DCEs: Discrete choice experiments
HIC: High-income countries
LMICs: Low and middle income countries
MMNL: Mixed multinomial logit
NCDIs: Non-communicable diseases and injuries
NIMAD: National institute for medical research development of Iran
RP: Revealed preference
SDGs: Sustainable development goals
SP: Stated preference
VSL: Value of statistical life
WTP: Willingness to pay
